# Differential Genome Size and Repetitive DNA Evolution in Diploid Species of *Melampodium* sect. *Melampodium* (Asteraceae)

**DOI:** 10.3389/fpls.2020.00362

**Published:** 2020-03-31

**Authors:** Jamie McCann, Jiří Macas, Petr Novák, Tod F. Stuessy, Jose L. Villaseñor, Hanna Weiss-Schneeweiss

**Affiliations:** ^1^Department of Botany and Biodiversity Research, University of Vienna, Vienna, Austria; ^2^Biology Centre, Czech Academy of Sciences, Institute of Plant Molecular Biology, České Budějovice, Czechia; ^3^Herbarium and Department of Evolution, Ecology and Organismal Biology, The Ohio State University, Columbus, OH, United States; ^4^Department of Botany, National Autonomous University of Mexico, Mexico City, Mexico

**Keywords:** ancestral state reconstruction, Bayesian analysis, genome size, *Melampodium*, phylogenetics, repetitive DNA, tandem repeats, transposable elements

## Abstract

Plant genomes vary greatly in composition and size mainly due to the diversity of repetitive DNAs and the inherent propensity for their amplification and removal from the host genome. Most studies addressing repeatome dynamics focus on model organisms, whereas few provide comprehensive investigations across the genomes of related taxa. Herein, we analyze the evolution of repeats of the 13 species in *Melampodium* sect. *Melampodium*, representing all but two of its diploid taxa, in a phylogenetic context. The investigated genomes range in size from 0.49 to 2.27 pg/1C (ca. 4.5-fold variation), despite having the same base chromosome number (*x* = 10) and very strong phylogenetic affinities. Phylogenetic analysis performed in BEAST and ancestral genome size reconstruction revealed mixed patterns of genome size increases and decreases across the group. High-throughput genome skimming and the RepeatExplorer pipeline were utilized to determine the repeat families responsible for the differences in observed genome sizes. Patterns of repeat evolution were found to be highly correlated with phylogenetic position, namely taxonomic series circumscription. Major differences found were in the abundances of the SIRE (*Ty1-copia*), Athila (*Ty3-gypsy*), and *CACTA* (DNA transposon) lineages. Additionally, several satellite DNA families were found to be highly group-specific, although their overall contribution to genome size variation was relatively small. Evolutionary changes in repetitive DNA composition and genome size were complex, with independent patterns of genome up- and downsizing throughout the evolution of the analyzed diploids. A model-based analysis of genome size and repetitive DNA composition revealed evidence for strong phylogenetic signal and differential evolutionary rates of major lineages of repeats in the diploid genomes.

## Introduction

Nuclear genome size is a strikingly variable characteristic of the flowering plants. Current estimates show more than 2000-fold genome size variation from the smallest known genomes of the carnivorous *Genlisea* (Lentibulariaceae; ca. 0.06 pg/1C) to the largest of *Paris japonica* (Melanthiaceae, 152.2 pg/1C) (Pellicer et al., 2010; Fleischmann et al., 2014). Although polyploidization, frequently occurring in plants, results in instant multiplication of the whole nuclear genome, differential evolution of the repetitive component of the genome explains the majority of the observed genome size variation in angiosperms (Bennetzen, 2005). However, the complexity of the repetitive fraction of the genome, both in terms of content and evolution and how it relates to species diversity, is still relatively unknown.

Repetitive DNA in plant genomes consists of two broad categories of repeat types, the dispersed mobile elements and tandem repeats (Bennetzen and Wang, 2014). Dispersed elements encompass mostly the DNA transposons and retrotransposons, which are commonly referred to as cut-and-paste and copy-and-paste elements, respectively. The long-terminal-repeat (LTR) retrotransposons are the most frequently occurring elements in most plant genomes and are typically distributed throughout the chromosomes. These repeats encompass several superfamilies, of which *Ty1-copia* and *Ty3-gypsy* are the most common in plants (Wicker et al., 2007). Which of these two superfamilies predominates in the genome can differ between plant groups/families (Novák et al., 2014; Kelly et al., 2015; Macas et al., 2015).

Tandem repeats include the ribosomal DNAs (5S and 35S) and satellite DNAs. In contrast to transposable elements, tandem repeats are typically found in distinct loci in the chromosomes. The ribosomal loci are the only known coding tandem repeats in the genome and can be localized in multiple places in the chromosomes. While both rDNA loci are useful phylogenetic and cytogenetic markers, 35S rDNA in particular is commonly used as a model for investigating processes of homogenization and epigenetic gene regulation (Kovařík et al., 2008; Zozomová-Lihová et al., 2014). Satellite DNAs are long arrays of repeated sequence monomers found in centromeric, telomeric and interstitial chromosomal regions of the genome. These repeats can make up a substantial portion of the plant genome (more than 30% in some species; olive – Barghini et al., 2014; *Frittilaria* – Kelly et al., 2015) and evolve quickly as evidenced by their high sequence variability among species (Melters et al., 2013; Garrido-Ramos, 2015). The latter phenomenon is explained by the library hypothesis, where the species of a related group contain different amounts of a common set of satellite repeats that are differentially amplified and might evolve independently after speciation (Ugarković and Plohl, 2002; Ugarković, 2008).

The extent and rate of amplification and removal of various repeats in the genome is the key driver of genome size evolution. Seed plant genomes, in general, contain more repetitive DNA than their animal counterparts, which makes them excellent systems for studying the evolution of repetitive DNA. Few comparative studies of the repeat landscape from closely related groups of organisms are available as of yet, as this has only recently become feasible with the advent of high-throughput sequencing (HTS) and the RepeatExplorer pipeline (Novák et al., 2010, 2013). This pipeline uses genome skimming data and graph-based clustering algorithms to identify repeat families (clusters) in the genome and calculate their abundances. Sequence reads from multiple closely related species can also be analyzed together in a comparative analysis, whereby individual clusters contain sequence reads from multiple species, indicative of the presence of homologous repeat families in their genomes (Macas et al., 2015).

The utility of RepeatExplorer and the comparative approach has been demonstrated in several study systems thus far, focusing on different phylogenetic scales (Novák et al., 2014; Macas et al., 2015; Vu et al., 2015) and allopolyploid systems (Renny-Byfield et al., 2013; Dodsworth et al., 2016). These studies have shown, in addition to characterizing repeats in the focal genomes, that the repeat family abundances are phylogenetically informative. An analysis conducted using maximum parsimony and likelihood approaches to reconstructing phylogenies from continuous characters in several different plant groups showed that these phylogenies gave similar results to those from commonly used molecular markers, with similar node support (Dodsworth et al., 2015).

A good system for investigating repetitive DNA evolution is the plant genus *Melampodium* (Asteraceae). which comprises 40 species distributed in Mexico. The largest section in the group, section *Melampodium* (*x* = 10), comprises 22 species, with 13 diploid species and nine exclusively polyploid or having both diploid and polyploid cytotypes (Blöch et al., 2009). All species have had their karyotypes established including localization of 5S and 35S rDNA loci and genome sizes measured (Weiss-Schneeweiss et al., 2009, 2012). Although all diploid species have the same chromosome number, they exhibit approximately 4.5-fold variation in genome size (0.49 pg–2.27 pg/1C) and are classified into five phylogenetically distinct series (Stuessy et al., 2011).

Herein, we investigate repetitive DNA and genome size evolution in all but two of the diploid species (13 of 15) in *Melampodium* sect. *Melampodium*. Material for *M.* s*inuatum* was not available, and the isolated phylogenetic position of *Melampodium longipilum* (series *Longipila*) relative to the other species of section *Melampodium* inferred using nuclear markers (Blöch et al., 2009; Stuessy et al., 2011; McCann et al., 2018) precluded it from analyses. The main goal of this study is to elucidate patterns of genome size change along the phylogeny of the group through an investigation of repetitive DNA evolution. Therefore, we extend the aforementioned results to test hypotheses that (1) genome size evolution has included both up- and downsizing, (2) repetitive DNA composition of these diploid genomes is strongly correlated with the phylogeny of the section, and (3) repeat lineages have different rates of evolution. To this end, ancestral genome size of *Melampodium* sect. *Melampodium* is reconstructed using traditional model-based approaches, the composition of repeats in the analyzed diploid genomes is characterized using the RepeatExplorer pipeline, and a novel model-based approach in a Bayesian framework is applied to reconstruct a phylogeny using comparative repeat abundances and to estimate rates of evolution for different repeat types.

## Materials and Methods

### Sequence Acquisition and Phylogenetic Analysis

Sequences of the internal transcribed spacer (ITS1-5.8S-ITS2) of 35S rDNA, the non-transcribed spacer (NTS) of 5S rDNA, two paralogs of the *phosphoglucoisomerase* gene (*PgiC*I and *PgiC*II) and the chloroplast gene *maturase* K (*mat*K) from all available diploid species in *Melampodium* sect. *Melampodium* published in earlier works (Blöch et al., 2009; Stuessy et al., 2011; Weiss-Schneeweiss et al., 2012) were used (summarized in Supplementary Table S1). Alignments were performed in Muscle 3.8.31 (Edgar, 2004) and further refined manually in Geneious R6 (Kearse et al., 2012).

Phylogenetic inference of species trees using all markers was performed using the StarBEAST2 package implemented in BEAST version 2.4.4 (Bouckaert et al., 2014; Ogilvie and Drummond, 2016). The overall rate of molecular evolution for each marker was inferred separately using the species tree uncorrelated relaxed log-normal clock model (Ogilvie and Drummond, 2016). The prior on the mean of the log-normal relaxed clock was log-normal with mean 0.005 and standard deviation 0.35 (in log space). The standard deviation prior was exponential with mean equal to 1/3. A log-normal calibration with mean 5.5 million years and standard deviation 0.23 in real space (McCann et al., 2018) was used on the root of the tree.

Four independent MCMCs were run for 500 million generations, with burn-in of 10% and sampling every 100K generations. The log files were all checked for convergence (Effective Sample size [ESS] > 200 for all parameters) using Tracer 1.6 (Rambaut, 2007). A maximum clade credibility (MCC) tree was calculated from the combined set of trees (1125 from each run, 4500 total), with mean node heights and no limit on the posterior probability of each clade.

### Ancestral Genome Size Reconstruction

Ancestral genome size was reconstructed using a set of twelve species, without *M. moctezumum* for which sequence data were not available. Reconstruction of ancestral genome size was performed on each tree in the post burn-in combined set from the BEAST runs (see above) in RevBayes, with the following three models: normal Brownian motion, Ornstein–Uhlenbeck, and a relaxed clock model. All three models shared a single rate parameter for genome size evolution. The latter two models, however, had an additional parameter allowing for either selection toward an optimum value or Brownian rate variation among the branches in the tree. A simple test of whether the estimate of these parameters was equal to zero was used to determine the validity of the model.

The data augmentation method was used for all models, and the script was adapted from the RevBayes tutorials (Horvilleur and Lartillot, 2014). This method allows reconstruction of the internal node states for each tree, with the need for prior specifications on the Brownian motion evolutionary rate and the root state of the tree. Wide uniform priors were placed on the logarithm of the Brownian motion rate (−5, 5) and the root genome size (−100, 100). These rate priors were used in all three analyses. For the Ornstein–Uhlenbeck model, the root genome size was used as the optimum value and the prior on the strength of selection was the same as that used for the Brownian motion rate. The mean of the log-normal for the relaxed clock analyses was set to 1 (as the Brownian motion rate was already estimated), and an exponential prior with mean 1/3 was used on the standard deviation.

For each tree and model a single MCMC run of 1 million generations with 100K burn-in was performed. Each run was checked for convergence (effective sample size > 200 of each model parameter) using custom python scripts. Posterior samples of ancestral genome sizes for all nodes in the MCC tree were extracted from the log files of the best model for each tree, provided the node was present (few nodes in the MCC had posterior support of 100%, see results), and combined into a single file for calculation of mean and 95% credible intervals using the coda R package (Plummer et al., 2006).

The program BayesTraits was used to estimate the δ, κ, and λ parameters, which are commonly used in ancestral state reconstructions to test for the presence of various evolutionary processes (Pagel and Meade, 2004). The δ parameter scales the tree in a way such that it can be detected if the rate of evolution of the trait in question changed as a function of distance from the root. The κ parameter also scales the branch lengths of the tree and lower values, for example, κ = 0, indicates that the branch lengths are not informative for the evolution of the trait. The λ parameter provides a measure of statistical independence of trait evolution and phylogeny, i.e., a value of 0 indicates that phylogenetic structure does not affect trait evolution. Four separate runs of BayesTraits were performed using the MCMC method of the program over the posterior set of trees. The different runs corresponded to independent estimates of each of the aforementioned parameters. The posterior distribution of these parameters was interpreted according to the BayesTraits manual (Pagel and Meade, 2004).

### DNA Isolation and High-Throughput Sequencing

Genomic DNAs were isolated for all diploid species or cytotypes in *Melampodium* sect. *Melampodium* with the exception of *M. sinuatum* (excluded due to lack of material) and libraries were prepared as outlined in McCann et al. (2018). Briefly, genomic DNA samples from two to three individuals were isolated and checked for quality and concentration using a NanoDrop spectrophotometer and Quant-iT PicoGreen dsDNA assay kit (PeqLab, Erlangen, Germany). DNA samples were pooled (species-wise) in equal proportions and fragmented to 600–800 nt in length. All samples were sequenced on a single lane of an Illumina HiSeq2500 machine using the 150 nt paired-end technology. Fragmentation, library preparation and sequencing were all performed at the CSF-NGS sequencing facility (Vienna Biocenter, Austria).

Read pre-processing, including quality filtering and removal of reads with similarity to the *PhiX* spike-in DNA (Illumina) or chloroplast sequences, was performed as outlined in McCann et al. (2018). *M. longipilum* was expected to be very different in nuclear DNA composition, due to incongruence in its phylogenetic position between nuclear and chloroplast markers (Blöch et al., 2009). Initial analyses confirmed extensive differences in repeat composition compared to other species and therefore *M. longipilum* was not included in the comparative analysis (see Discussion). The reads were analyzed using the command line implementation of the RepeatExplorer pipeline with the default settings and using the maximum number of reads possible with 100 GB of RAM. Briefly, the RepeatExplorer performs an all-to-all blast comparison and clusters sequence reads based on similarity (Novák et al., 2010, 2013). The clusters containing a minimum of 0.01% of the total reads used were annotated using BLAST searches to manually curated transposable element domain databases, graph structure, dot-plot structure (Sonnhammer and Durbin, 1995) and paired-end read connections (Macas et al., 2015).

A comparative analysis was also performed where forward ends of paired-end reads used in the individual analyses were randomly sampled proportional to each species genome size and pooled into a single dataset. The use of single-end reads increased the randomness of each sample of genome, whereas using reads from the individual analyses allowed for automated annotation of the comparative analysis clusters by tracing reads back to clusters of their origin. The settings for the comparative analysis were essentially the same as those of the individual species. The analyses were repeated three times to check for the congruency of the results.

The number of reads from each species was quantified for each repeat cluster and used to compare the abundance of each cluster across all species. Hs/Ho ratios were calculated for each read in the comparative analysis as outlined in Macas et al. (2015). This statistic is the ratio of the frequency of BLAST hits to reads from the same species (*Hs*) to the frequency of hits to reads from all other species (*Ho*). The ratio was calculated for *Ho* where only species within the same series are included and for all other species regardless of serial classification.

### Phylogeny Reconstruction Using Repeat Abundances

Genomic repeat abundances obtained from annotated repeats in the comparative analysis were cube-root transformed and used as continuous characters to reconstruct the phylogeny of section *Melampodium* using the program RevBayes (Höhna et al., 2016), developed specifically for phylogenetic inference. The method of phylogenetic independent contrasts using restricted maximum likelihood (Felsenstein, 1985) was applied to reconstruct phylogenies using several different model specifications with varying complexity. All analyses were adapted from scripts provided in the RevBayes tutorials.

Common to all models, priors on the diversification and turnover rates were chosen as follows: a log-normal distribution with mean 0 and standard deviation 1 (in real space) and a gamma distribution with shape and rate parameters equal to 4. Each model specification differed only in the number of rates estimated across the cluster abundance data matrix. Theoretically, this number could range from one, where all repeats evolve with the same rate, to the total number of repeat clusters analyzed, which assigns an independent rate parameter to each cluster.

In addition to single rate and all independent rate model specifications, a number of intermediate alternative models were also tested. These models imposed various constraints on the rates of evolution for different repeat types. For example, one model allowed different repeat types to evolve at different rates, but within a single type the rate of evolution was forced to be the same. Some within-repeat type variation was allowed, especially in satellite DNA repeats, where clusters within a particular repeat type are likely to have evolved differently among species. Each rate in all model specifications was assigned a wide uniform prior ranging from −5 to 5 in log space. Stepping stone sampling was performed to estimate the marginal likelihoods for each model. The ratio (or difference if log-transformed) of the marginal likelihoods of any two models gives the Bayes factor, which is a metric commonly used for scoring relative model fit. The marginal likelihoods were calculated for each model and Bayes Factors were calculated for the top models. The best model was used for final tree inference and interpretation of patterns of repeat evolution in *Melampodium*.

## Results

### Phylogenetic Inference Using Sequence Data in BEAST

**FIGURE 1:**
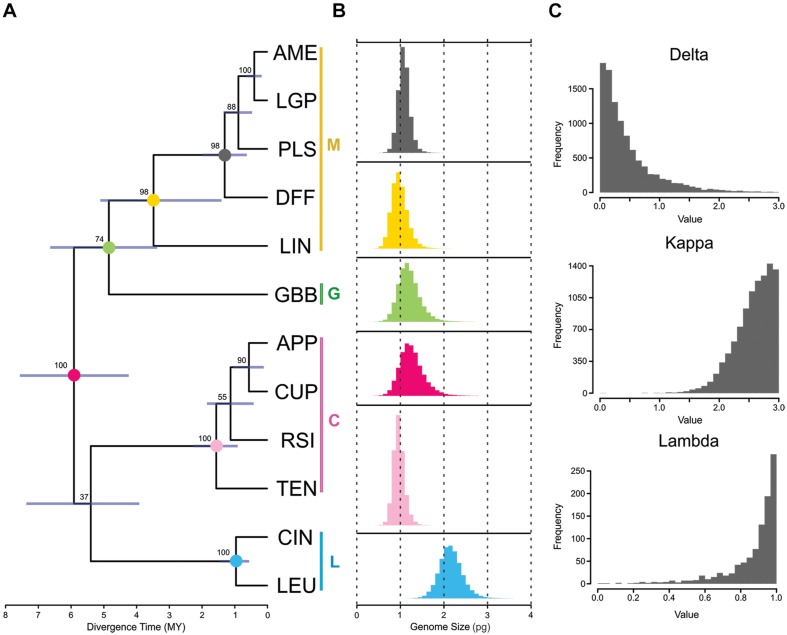
Results of phylogenetic inference and ancestral genome size reconstruction in section *Melampodium*: **(A)** the maximum clade credibility tree from the BEAST analysis; Color vertical lines and letters next to species names indicate the taxonomic series the species belong to (C, series *Cupulata*, pink; G, series *Glabribracteata*, green; L, series *Leucantha*, blue; M, series *Melampodium*, yellow). **(B)** posterior distributions obtained from RevBayes analysis of ancestral genome size on important, well-supported nodes [annotated by color at nodes in **(A)**]; and **(C)** posterior distributions of commonly used tree transformation statistics (δ, κ, and λ) in BayesTraits. The light-blue bar at each node indicates uncertainty in the node height of the tree.

The phylogenetic tree for twelve diploid species in *Melampodium* sect. *Melampodium* was inferred using the multi-species coalescent in BEAST and the resulting MCC tree for the multi-species coalescent inference in BEAST is shown in Figure 1A. Posterior nodal support across the tree was generally quite high, with clade support ranging from 37 to 100%. The clades representing the different series in section *Melampodium* had very high support (97–100%). The least well-supported node (37%) was the split joining series *Cupulata* with *Leucantha*. Additionally, the phylogenetic position of *Melampodium glabribracteatum* as sister to series *Melampodium* only had 74% support. This low certainty left the backbone of the phylogeny of section *Melampodium* unresolved (Figure 1A).

### Ancestral Genome Size Reconstruction

Three different models of genome size evolution were tested on all trees obtained from the BEAST phylogenetic analyses. The simple Brownian motion model only had a single parameter to estimate, namely the rate of genome size evolution. The other two models, the Ornstein–Uhlenbeck and relaxed rate model, had one additional parameter each, namely parameters for the strength of selection toward the optimum value and the standard deviation of the log-normal rate distribution across the tree, respectively. For all trees, these three models reduced to simple Brownian motion, where the aforementioned parameters were estimated to be essentially equal to zero. Therefore, the simple Brownian motion model was considered the best evolutionary model for genome size evolution in section *Melampodium*.

The posterior distribution of ancestral genome sizes from the Brownian motion analysis for well-supported nodes in the maximum clade credibility tree are shown in Figures 1A,B. DNA sequence data from *Melampodium moctezumum* were not available for use in phylogenetic inference, and hence, it was not used for ancestral genome size inference. The mean genome size reconstructed for the ancestor of the entire group was 1.27 pg/1C with highest-posterior density (HPD) of 0.81–1.72. The mean values of genome size reconstructions for the individual series in section *Melampodium* were similar to the observed genome size ranges for the species they contained except for series *Melampodium*. This series contained *M. linearilobum*, which has a much smaller genome size than the other species in the group (Table 1). The mean of the posterior distribution for the whole group was 0.98 pg/1C, with HPD ranging from 0.68 to 1.28. The clade containing series *Melampodium* and the monotypic series *Glabribracteata* (1.89 pg/1C) had a mean of 1.20 pg/1C and HPD range of 0.79–1.59 (Figures 1A,B).

**TABLE 1:** General information about the analyzed species as well as the individual and comparative clustering analyses of high-throughput sequencing (HTS) reads using the RepeatExplorer bioinformatic pipeline.

**Species**	**ID**	Genome Size*	**Individual clustering**		**Comparative clustering**
		**Gbp/1C**	**No. reads**	**Coverage**	**No. reads**
**Series *Melampodium***					
*M. americanum*	AME	1.11	7621416	0.89x	897117
*M. diffusum*	DFF	1.07	7797006	0.95x	888532
*M. linearilobum*	LIN	0.48	7957510	2.13x	388729
*M. longipes*	LGP	1.11	6736416	0.79x	880500
*M. pilosum*	PLS	1.07	7889718	0.96x	833622
**Series *Glabribracteata***					
*M. glabribracteatum*	GBB	1.81	5555528	0.40x	1465723
**Series *Cupulata***					
*M. appendiculatum*	APP	0.92	7522676	1.06x	753557
*M. cupulatum*	CUP	0.92	7327880	1.04x	738084
*M. moctezumum*	MZT	0.98	5946104	0.96x	737049
*M. rosei*	RSI	0.92	5787404	0.82x	721808
*M. tenellum*	TEN	0.92	4568954	0.65x	729206
**Series *Leucantha***					
*M. cinereum*	CIN	2.17	7560312	0.45x	1761046
*M. leucanthum*	LEU	2.27	8062040	0.46x	1840574

* Genome sizes taken from Weiss-Schneeweiss et al. (2012) and Rebernig et al. (2012).

The program BayesTraits was used to estimate δ, κ, and λ, which are tree transformations that provide information about rates of evolution and inform if the particular trait appears to evolve independently of phylogenetic tree structure. The distribution of δ was strongly skewed toward 0 with a 95% HPD interval of 0.001–1.457 (Figure 1C). The distribution of κ, which has the same range of possible values as δ, was strongly skewed toward the other end of the range (3) with a 95% HPD of 1.976–3.0. The λ parameter, ranging between possible values of 0 and 1, had a 95% HPD interval of 0.543–1.0 (Figure 1C), indicating high phylogenetic signal in the genome size data.

### Repetitive DNA Content of *Melampodium* Genomes

The *Melampodium* species analyzed using the RepeatExplorer pipeline are shown in Table 1. The genome sizes were published in previous studies (Table 1). Clustering of individual species reads was performed on a number of reads corresponding to between 0.40 and 2.13x coverage of the genome for each species (Table 1). Approximately 46–70% of the reads in each individual species analysis were found in medium to high copy number clusters comprising at least 0.05% of their genomes. Post-processing and further analysis allowed for the assignment of 90–96% of these clusters to specific repeat types, with the remainder left unannotated (Table 2). This amounted to approximately 40–64% of the total genome being annotated. Overall repeat composition in section *Melampodium* largely corresponded with the classification of the group at the series level. The genomic proportions of different repeat types observed in the analyzed genomes were summarized according to their taxonomic classification in Table 2.

**TABLE 2:** Genome proportion estimates (%) from the individual RepeatExplorer analyses of repeats found in the genomes of the diploid species in *Melampodium* sect.

**Type**	**Lineage**	***Cupulata***	***Glabribracteata***	***Leucantha***	***Melampodium***
Retrotransposons	–*	32.5–48.6	59.7	54.1–55.1	38.5–49.8
*Ty1-copia*	–*	19.5–28.0	37.4	27.8–30.0	18.9–24.5
	SIRE	18.1–26.4	35.7	24.9–27.5	17.3–22.7
	Other	1.4–1.7	1.7	2.6–2.9	1.2–1.7
*Ty3-gypsy*	–*	11.7–17.4	21.0	20.2–23.4	19.0–23.5
	Athila	2.8–4.9	10.1	7.4–10.4	8.3–9.9
	Chromo	5.2–7.4	7.4	7.3–8.0	6.7–8.2
	Ogre/Tat	2.9–5.4	3.3	4.3–4.4	4.0–6.0
Other/Non-LTR	–	0.4–1.6	0.7	0.6–0.7	0.4–0.7
	Pararetrovirus	0.1–0.2	0.3	0.2–0.3	0.1–0.2
	LINE	0.2–0.7	0.2	0.2–0.2	0.0–0.1
	Other	0.1–0.7	0.2	0.2–0.2	0.3–0.4
DNA transposons	–	0.6–1.9	2.8	1.1–1.4	1.0–4.5
	*CACTA*	0.2–0.9	2.4	0.2–0.3	0.6–3.6
	Other	0.4–1.0	0.4	0.9–1.1	0.4–0.9
Tandem repeats	–	4.2–8.1	1.3	1.2–1.7	2.0–4.4
	35S rDNA	0.9–3.0	1.0	0.5–0.6	0.8–2.0
	5S rDNA	0.1–0.2	0.1	0.1–0.1	0.1–0.1
	Satellite DNAs	3.2–4.9	0.2	0.7–1.0	1.3–2.3
Unclassified	–	4.0–5.8	5.3	6.3–6.6	7.9–10.0
Low-Copy	–	37.9–54.1	29.8	34.7–34.8	35.4–43.5

*Melampodium* grouped in series. Ranges are given for series with more than one species. * Total content of a given type including LTR-retrotransposons not assigned to either a superfamily or specific lineage within the superfamily.

Long-terminal-repeat-retrotransposons constituted the largest proportion of all analyzed *Melampodium* genomes, reaching up to nearly 60% in *M. glabribracteatum*. Nearly equal proportions of *Ty1-copia* and *Ty3-gypsy* elements were found in most species, with the exception of those in series *Glabribracteata* and *Leucantha*. These series had significantly higher amounts of *Ty1-copia* relative to *Ty3-gypsy* elements (nearly 3:1 in *M. glabribracteatum*) than the remaining series (Table 2). SIRE repeats made up the vast majority of the *Ty1-copia* elements (18–36%), while the other *Ty1-copia* lineages combined represented <3% of the analyzed genomes. The genomic proportions of the three lineages of the *Ty3-gypsy* superfamily (Athila, Chromovirus, Ogre-Tat) were more balanced, although the Athila lineage was typically the most common (Table 2). Pararetroviruses, LINE, SINE, and MITE elements were found only in trace amounts in *Melampodium*.

Most of the lineages of DNA transposons and the Helitrons were represented in *Melampodium* genomes, albeit mostly in small amounts (Table 2). Of these lineages, *CACTA* elements were typically the most abundant, reaching genome proportions of 0.2 up to 6.6% of the genome in series *Cupulata* and in *M. linearilobum* of series *Melampodium*, respectively. The Helitrons and other DNA transposon lineages were found only in trace amounts across all species in section *Melampodium*.

Tandem repeats (5S rDNA, 35S rDNA, and satellite DNAs) were found in relatively low abundance in all species. Both the 35S and 5S rRNA genes ranged from 0.5 to 3% and 0.1 to 0.2% in the genome, respectively (Table 2). The number of satellite DNAs observed across all genomes was relatively low. Satellite DNAs were found in proportions as low as 0.5% in *M. glabribracteatum* and approaching 5% in some species of the *Cupulata* group.

### Dynamics of Shared Repeats Across Section *Melampodium*

The comparative analysis of the repetitive DNA fraction entailed clustering of reads from different species to identify repeats that were shared or species/group specific across all genomes. This was performed using reads from all diploid species in section *Melampodium* listed in Table 1. The total number of reads analyzed per species corresponded to 0.1x coverage of each of their genomes (Table 1). These reads were clustered together and resulted in 438 clusters comprising at least 0.05% of the total number of reads analyzed.

**FIGURE 2:**
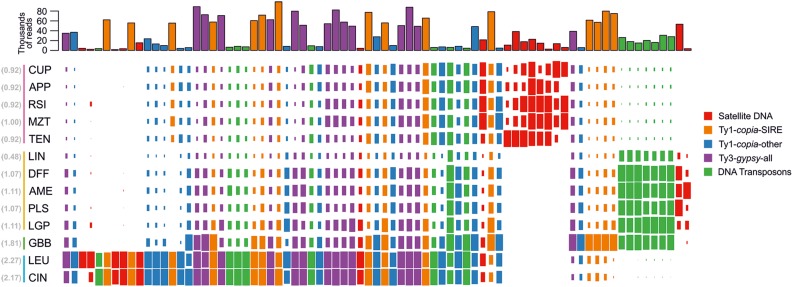
Normalized genome representation of the top clusters of five major repeat groups found in the genomes of species analyzed in the comparative analysis. Each of the four major repeat types (Ty1-*copia*-SIRE; Ty1-*copia*-others; Ty3-*gypsy*-all and DNA transposon) is represented by 15 top clusters whereas satellite DNA is represented by all 17 clusters. The repeats are hierarchically clustered along the horizontal axis so that repeats with similar distributions across species are grouped together. Genome size (1C value) for each species is indicated next to species name abbreviation (in brackets, gray) and the color vertical lines to the left of the species names indicate the taxonomic series the species belong to (series *Cupulata*, pink; series *Glabribracteata*, green; series *Leucantha*, blue; series *Melampodium*, yellow).

The top 438 clusters exhibited wide variation in the proportion of reads from individual species, ranging from clusters containing reads from all species to clusters composed of reads from multiple species in a single taxonomic series (Figure 2). Overall, the majority of clusters contained reads from all species, where read abundance for each species was proportional to their genome sizes. The distribution of reads from LTR-retrotransposons mostly followed this trend, with the exception of clusters annotated as *Ty1-copia* SIRE lineage. These repeats exhibited both proportional distribution of species’ reads along with several clusters being specific to either *M. glabribracteatum* or series *Leucantha*. On the other hand, the vast majority of repeat clusters in the *Ty3-gypsy* superfamily and *Ty1-copia* lineages other than SIRE were shared across all species. The DNA transposons displayed similar patterns to clusters of *Ty1-copia* type, with one lineage (*CACTA*) showing series-specificity and other lineages containing reads from all species.

**FIGURE 3:**
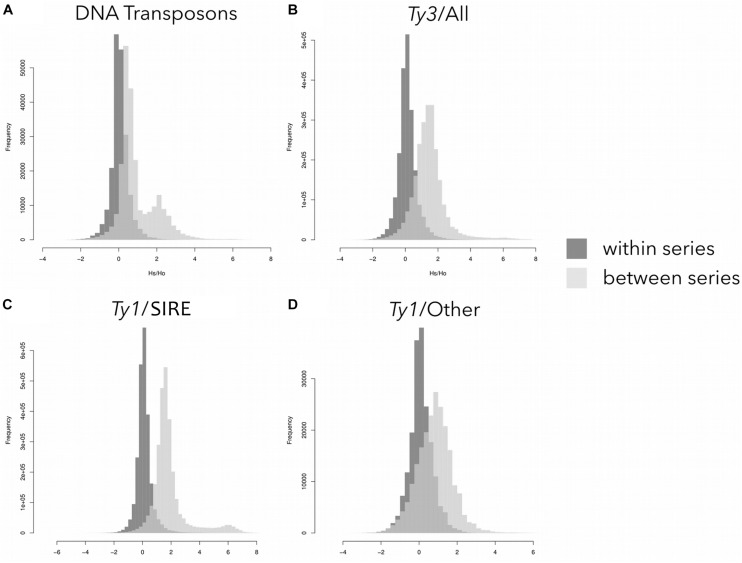
Sequence similarity profiles (distributions of the logarithm of Hs/Ho) for different repeat types: **(A)** DNA transposons; **(B)** Ty3-*gypsy* elements; **(C)** Ty1-*copia* SIRE elements; and **(D)** other Ty1-*copia* elements except for the SIRE lineage. For each read the Hs/Ho ratio was calculated using either hits to reads from species in the same series (dark gray) or hits to reads from all other species (light gray).

Sequence similarity profiles (*Hs/Ho* distributions) were calculated for both within-series and between-series comparisons. The within-series comparisons revealed single peaks centered around zero for all major types of transposons (Figure 3). Between-series comparisons also showed single peaks for all *Ty3-gypsy* and *Ty1-copia* elements not derived from the SIRE lineage, albeit with means located around 1.5 (Figures 3B,D). The DNA transposons and *Ty1-copia* SIRE elements had between-series *Hs/Ho* distributions with secondary peaks shifted to the right (Figures 3A,C). The secondary peaks for the DNA transposons and *Ty1-copia* SIRE retroelements were centered around 2 and 6, respectively, indicating significant differences in similarities in this repeat type among groups.

**FIGURE 4:**
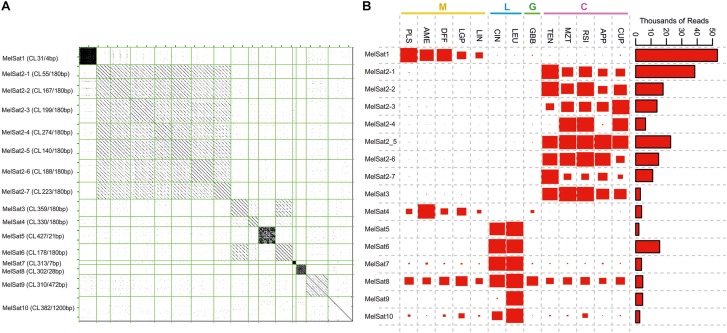
A comparison of satellite DNAs identified in the comparative analysis of all species analyzed in this study: **(A)** Dot-plot comparison of identified satellite DNA clusters (each represented by one contig) and **(B)** the contribution of reads from different species to each satellite DNA cluster. The area of red rectangles is proportional to the number of reads (abundance) contributed by each species. Color bars and capital letters above species names indicate the taxonomic series the species belong to (C, series *Cupulata*, pink; G, series *Glabribracteata*, green; L, series *Leucantha*, blue; M, series *Melampodium*, yellow).

Most of the satellite DNAs identified in the comparative analysis dataset exhibited a high degree of series-specificity in section *Melampodium* (Figure 4B). Eight satellite DNA clusters were specific to the series *Cupulata* alone (Figure 4). Seven of the eight satellite DNAs found in this series were highly similar both in monomer length and sequence composition, while the eighth (MelSat3, Figure 4A) was similar to a satellite found also in the *Leucantha* series (MelSat6, Figure 4A). Several unique, series-specific satellite DNA clusters were also found in series *Leucantha*, although, unlike in the *Cupulata* group, monomer sequences showed no similarity to one another. One 28 nt satellite monomer was recovered in all 13 analyzed species (MelSat8, Figure 4B). The majority of the monomer lengths in these species were approximately 180 nt in length, but ranged from 4 to 1200 nt. One cluster, which was recovered mostly from the *Leucantha* series (MelSat7, Figure 4B), contained perfect 7 nt telomeric repeats (TTTAGGG).

### Phylogenetic Support for Independent Evolution of Repeat Types in Section *Melampodium*

Repetitive DNA evolution in *Melampodium* was further analyzed by reconstructing the phylogeny of the section using repeat abundances from the comparative analysis. These abundances were treated as continuous characters and used to construct phylogenies under several Brownian motion models of evolution with varying levels of complexity (i.e., number of rate parameters). Model selection was performed to determine which model best fits the data, thus providing information about which repeats evolve at similar rates.

Marginal likelihood estimates (MLE) were calculated using stepping stone sampling and ranged from −6954.64 to −42777 (Table 3). The simplest and most complex models had the lowest MLEs, while a model with seven rate parameters performed the best. This model applied a single unique rate to each of the following groups: DNA transposons, LINE, *Ty1-copia* SIRE, non-SIRE *Ty1-copia*, *Ty3-gypsy*, and tandem repeats (including rDNA repeats). The remaining repeat types were grouped together into a single rate class.

**TABLE 3:** Marginal likelihood estimates (MLE) for all models used to construct repeat phylogenies.

**Model**	**1**	**2**	**3**	**4**	**5**	**6**	**7**	**8**	**9**	**10**
No. parameters	1	6	7	19	23	24	24	25	40	307
MLE	−7284.09	−7030.16	−6954.64	−7074.39	−6971.97	−7005.63	−7058.18	−6984.70	−7055.72	−42777

*Full model specifications are available from the authors upon request.*

**FIGURE 5:**
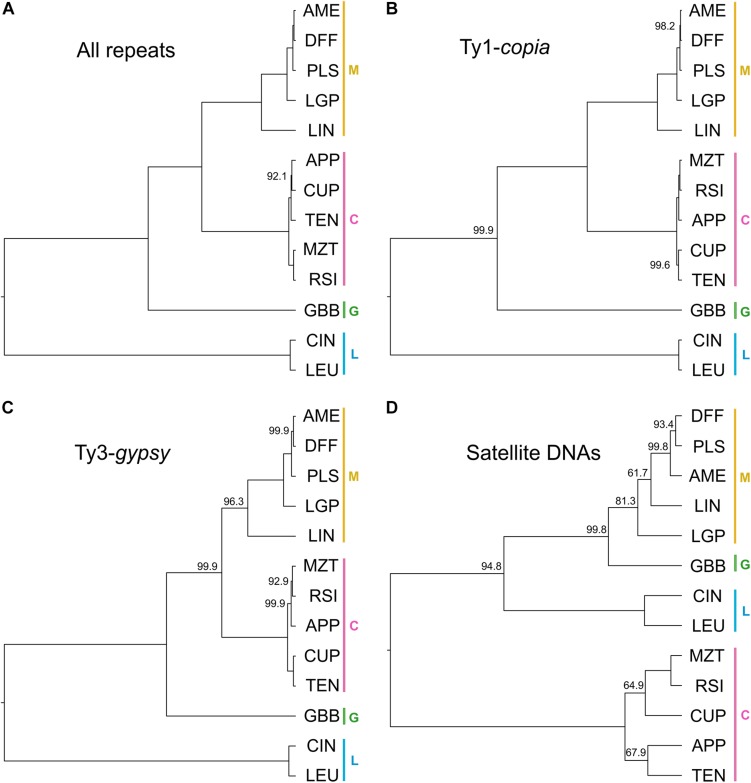
Maximum *a posteriori* trees recovered from repeat-abundance based phylogenetic analysis with a single rate parameter and fixed root height of 1: **(A)** all repeats included with best model; **(B)** Ty1-*copia* elements only; **(C)** Ty3-*gypsy* elements only; and **(D)** satellite DNAs only. Color bars and capital letters next to species names indicate the taxonomic series the species belong to (C, series *Cupulata*, pink; G, series *Glabribracteata*, green; L, series *Leucantha*, blue; M, series *Melampodium*, yellow).

The relationships recovered from the phylogenetic analysis using repeat abundances (with the best supported model) as characters are shown in Figure 5A. In general, the posterior support in this analysis was higher than that from the BEAST analysis. The node posterior support across the entire tree was always greater than 90% and in most cases was 100% supported. The serial classification within section *Melampodium* was well-supported in this analysis. The maximum *a posteriori* (MAP) tree placed series *Melampodium* and *Cupulata* as sister groups, whereas *M. glabribracteatum* was recovered as sister to those groups. Series *Leucantha* was sister to all others.

To investigate differences in topology and support, phylogenetic analyses were performed for a few of the major repeat types separately. Maximum *a posteriori* (MAP) trees were constructed for each analysis (Figures 5B–D). The two MAP trees derived from analyses of *Ty1-copia* or *Ty1-gypsy* elements (Figures 5B,C, respectively) showed similar topology including their branch lengths, with slightly weaker support in the *Ty3-gypsy* tree moving toward the tips of the tree. The satellite DNA tree topology (Figure 5D), however, had overall much weaker node support, larger differences in branch lengths and well-supported topological differences from both LTR-retrotransposon based trees and the tree based on all repeats.

## Discussion

The diversity of repetitive DNAs and the inherent propensity for their amplification and removal from the host genome greatly contributes to the variation of plant genome sizes and composition. This study addresses repeatome dynamics across the genomes of 13 closely related wild diploid species of genus *Melampodium* that differ nearly 4.5-fold in genome size despite having the same base chromosome number (*x* = 10). Evolutionary changes in repetitive DNA composition and genome size were complex, with independent patterns of genome up and downsizing throughout the evolution of the analyzed diploids mostly due to changes in abundances of the SIRE (*Ty1-copia*), Athila (*Ty3-gypsy*), and *CACTA* (DNA transposon) lineages. Evidence for strong phylogenetic signal and differential evolutionary rates of major lineages of repeats in the diploid genomes of *Melampodium* was also inferred.

### Genome Size Evolution

Genome size values for diploid species in section *Melampodium* range from 0.49 to 2.27 pg/1C (Rebernig et al., 2012; Weiss-Schneeweiss et al., 2012). The range of genome sizes for all species in section *Melampodium* are below average relative to other species in Asteraceae (2.91 ± 1.22, Vallès et al., 2013). Patterns of genome size variation in section *Melampodium* follow the serial classification and are, thus, congruent with phylogenetic position of taxa. The only exception is *M. linearilobum*, which has reduced genome size relative to the other species in the same series (*Melampodium*).

The correlation between species-relatedness and genome size observed in section *Melampodium* suggests that this character contains phylogenetic signal. Statistical support for this finding was provided by the estimation of *Pagel’s*λ, which demonstrated the dependence of genome size on the internal structure of the tree. The strong phylogenetic signal of genome size in section *Melampodium* and lack of support for the Ornstein–Uhlenbeck model provides evidence against processes that could erode this signal, such as selection toward a single optimum genome size (Bloomberg et al., 2003; Harmon et al., 2010). Estimates of the other two commonly used statistics, κ and δ, revealed that most evolution of genome size has occurred early on in the phylogeny and along longer branches (Pagel and Meade, 2004), supporting adaptive radiation and gradualism in the group, respectively. A punctuational hypothesis of genome size evolution has been supported in other plant groups, through either arguments against the correlation of sequence substitution rates and genome size in evolution or via statistical approaches similar to that applied in this study (Albach and Greilhuber, 2004; Lysak et al., 2009). While trees with branch lengths proportional to average substitutions per site may not be the best models for inferring patterns of genome size evolution, this can be ameliorated through the use of ultrametric trees, where branch lengths are proportional to time. Such an alternative approach circumvents the need for a strong *a priori* assumption that genome size evolves either independent of branch length or correlated with substitution rates and can instead be tested in a statistical framework (Cusimano and Renner, 2014; McCann et al., 2016).

Independence of character evolution and branch length implies punctuational change along the phylogeny (Lysak et al., 2009). In plants, such changes in genome size can be the result of either polyploidization or transposable element activity (Bennetzen and Wang, 2014). In the present analysis, only diploid lineages were analyzed therefore eliminating the need to consider recent polyploidy as a potential explanation for the observed genome sizes. Estimates of κ for section *Melampodium* suggest gradual evolution of genome size on longer branches in the phylogeny, as has been reported in Brassicaceae (Lysak et al., 2009). Branches leading to lineages with the largest differences in genome size (i.e., *M. glabribracteatum, M. linearilobum* and the two species in series *Leucantha*) are also the longest in the tree, suggesting more gradual changes of genome size in these species over time. Alternatively, punctuational increase of transposable element activity early on along long branches could also explain the observed genome size patterns. However, a model allowing for rate heterogeneity among branches (relaxed-rate model) in the tree was not supported over standard, constant-rate Brownian motion.

### Evolution of Repetitive DNA

Quantification of the contribution of various elements to the repetitive portion of diploid genomes in section *Melampodium* was performed using the RepeatExplorer pipeline, the utility and accuracy of which has been demonstrated in multiple previous studies (Novák et al., 2010, 2014; Macas et al., 2015). All but two of the diploid species of section *Melampodium* have been analyzed in this study. Material for *M.* s*inuatum* was not available. *M. longipilum* (series *Longipila*) has been sequenced and was analyzed individually. This analysis revealed significant differences in repeat composition relative to the other species in section *Melampodium*, which is congruent with its phylogenetic position using nuclear markers, where it groups outside of section *Melampodium* in non-sister group relation (Blöch et al., 2009; Stuessy et al., 2011; McCann et al., 2018). Thus, it was not included in the comparative analysis.

The repetitive fraction of *Melampodium* genomes ranged from 46 to 70% with larger genomes having correspondingly higher amounts of repeats. This range is within the estimates published for other species including a close relative *Helianthus annuus* (>3.0 pg/1C and 81% repetitive; Natali et al., 2013). The abundance of the various repeat types in plant genomes is largely group-specific and responsible for genome size evolution. For example, *Ty3-gypsy* elements were found to be most abundant in both *Gossypium* and species in Fabeae (Hawkins et al., 2009; Macas et al., 2015), and were inferred to play a role in genome size differentiation. In *Melampodium*, the SIRE lineage of *Ty1-copia* superfamily was largely responsible for most of the observed genome size increases, as the larger genomes of *M. glabribracteatum* and the two species in series *Leucantha* have higher amounts of these elements. However, *Ty1-copia* SIRE elements do not play a significant role in the genome downsizing observed in *M. linearilobum*. The genome of *M. linearilobum*, although strongly reduced in size, contains repeats in similar proportions to those found in the other species in the same series. In conjunction with the ancestral genome size analysis, this suggests proportional decrease of all repeat types in *M. linearilobum*.

The comparative analysis conducted in this study is one of the few to have near complete sampling of a group of related species, which allows for a better understanding of repetitive DNA evolution in a closely related group of species. This analysis confirmed the differential amplification of *Ty1-copia* SIRE lineages by revealing repeat clusters of SIRE type with higher abundances in *M. glabribracteatum* and the two species in the *Leucantha* series. Sequence similarity profiles (*Hs*/*Ho* distributions) showed significant secondary peaks in read comparisons among taxonomic series for reads derived from SIRE repeats, suggesting that similarity among these elements differs between groups. The results of ancestral genome size reconstruction and repetitive DNA analyses together provide evidence for continuous amplification of various subtypes of SIRE retroelements over time in section *Melampodium*, whereas amplification of specific repeat types has been shown to be punctuated in other plant groups (Hawkins et al., 2009; Belyayev, 2014; Macas et al., 2015). The SIRE lineage, thus, likely represents a recently active retroelement type in section *Melampodium*.

DNA transposon composition also differed across the genomes of species in section *Melampodium*. However, the lower overall genome proportion of these elements, relative to retrotransposons, suggests a limited role in genome size differentiation. A secondary peak in the sequence similarity profile (*Hs*/*Ho* distribution) for DNA transposons was found, although it was not as pronounced as that of *Ty1-copia* SIRE elements. This secondary peak was likely the result of increased amount of DNA transposons in the species of series *Melampodium* and *Glabribracteata*.

Satellite DNAs can arise from any DNA sequence in the genome and such repeats typically exhibit high rates of evolution both in monomer sequence and overall abundance (Tek et al., 2005; Macas et al., 2010; Melters et al., 2013). Several satellite DNA repeats were recovered in the comparative analysis and these exhibited the highest taxonomic specificity. Monomer length of satellite DNA repeats found in section *Melampodium* ranged from a 4 nt microsatellite (ATTC) to 1200 nt. However, the most frequently occurring monomer length found among the satellite DNAs was around 180 bases. This fits a common pattern found in satellite DNAs in that monomer lengths around 165 nt appear to be favored, which may be a consequence of structural constraints imposed by the nucleosome (Macas et al., 2002; Garrido-Ramos, 2015). Higher copy numbers of telomeric sequences recovered in *M. leucanthum* and *M. cinereum* might be caused by technical constrains either during the DNA extraction or DNA sequencing or, alternatively, might suggest the presence of longer telomeres in these two species.

Several satellite DNA repeat clusters were shared by all species in series *Cupulata*, nearly all of which had the same monomer length and highly similar monomer sequences. There is no indication of species-specificity for monomers of individual clusters, as most are shared among all series *Cupulata* species. Given such a pattern, these different satellite DNA monomers are likely descendants of one common repeat. Inter-chromosomal divergence and specificity of centromeric satellites has been observed previously in other study systems such as *Oryza* (Macas et al., 2010), *Arabidopsis* (Heslop-Harrison et al., 1999), and in humans (Rudd et al., 2006), suggesting that homogenization of centromere-specific satellite DNA repeats occurs mostly within individual chromosomes with low rates of inter-chromosomal spread. The chromosomal localization of these satellite DNA repeats in series *Cupulata*, however, has not yet been determined.

### Phylogenetic Signal in the Evolution of Repeats

The utility of repeat abundances in the genomes has been shown to allow reliable reconstruction of the phylogeny of a related group of species using both maximum parsimony and likelihood methods (Dodsworth et al., 2015). The program used for maximum likelihood estimation of a phylogeny (Felsenstein, 1993) assumes that each character evolves independently. However, this may lead to model overfitting as repeat clusters stemming from the same lineage may have correlated evolutionary histories.

In this study, we relaxed the assumption of independent rates of evolution and performed model testing in a Bayesian framework to reduce the number of rate parameters in the model, and thus reduce overfitting the model to the data. Our results show that neither the simplest or most complex models (single rate or all independent rates, respectively) fit the data as well as models restricting rates based on repeat annotation, although models with fewer parameters generally perform better.

Repeat clusters of different origin can provide variable levels of phylogenetic signal, therefore affecting tree topology and resolution (Dodsworth et al., 2015; Weiss-Schneeweiss et al., 2015). Our comparative analysis and model testing, as well as analysis of repeats in many other genomes (Renny-Byfield et al., 2012; Dodsworth et al., 2015; Macas et al., 2015), show that the repetitive landscape of genome does not evolve in a uniform fashion. Satellite DNA repeats may be expected to resolve relationships closer to the tips of the tree due to their high rates of evolution. Other repeats, such as retroelements, may provide better resolution toward the root, as they have persisted in the genome over longer evolutionary time. Phylogeny reconstruction using repeats derived from the *Ty1-copia* and *Ty3-gypsy* superfamilies agreed in overall topology and branch lengths, although the *Ty1-copia* tree had higher posterior support. The phylogeny obtained from abundances of satellite DNAs had much lower support and a different topology. These findings demonstrate that the levels of phylogenetic signal are dependent on repeat type and can lead to different topologies with varying levels of support.

## Data Availability Statement

The data generated by this study can be found in the European Nucleotide Archive (ENA) using accession number PRJEB36721 (ERP119943).

## Author Contributions

HW-S, JMc, and TS conceived and coordinated the study. JMc performed the research. JMc, HW-S, JMa, and PN analyzed the data. HW-S, JMc, JMa, and PN interpreted the data and wrote the manuscript with input from TS and JV. All authors read and approved the final manuscript.

## Conflict of Interest

The authors declare that the research was conducted in the absence of any commercial or financial relationships that could be construed as a potential conflict of interest.

## References

[B1] Albach D. C. Greilhuber J. (2004). Genome size variation and evolution in *Veronica*. *Ann. Bot.* 94 897–911. 10.1093/aob/mch219 15520022 PMC4242286

[B2] Barghini E. Natali L. Cossu R. M. Giordani T. Pindo M. Cattonaro F. (2014). The peculiar landscape of repetitive sequences in the olive (*Olea europaea* L.) genome. *Genome Biol. Evol.* 6 776–791. 10.1093/gbe/evu058 24671744 PMC4007544

[B3] Belyayev A. (2014). Bursts of transposable elements as an evolutionary driving force. *J. Evol. Biol.* 27 2573–2584. 10.1111/jeb.12513 25290698

[B4] Bennetzen J. L. (2005). Mechanisms of recent genome size variation in flowering plants. *Ann. Bot.* 95 127–132. 10.1093/aob/mci008 15596462 PMC4246713

[B5] Bennetzen J. L. Wang H. (2014). The contributions of transposable elements to the structure, function, and evolution of plant genomes. *Annu. Rev. Plant Biol.* 65 505–530. 10.1146/annurev-arplant-050213-035811 24579996

[B6] Blöch C. Weiss-Schneeweiss H. Schneeweiss G. M. Barfuss M. H. Rebernig C. A. Villaseñor J. L. (2009). Molecular phylogenetic analyses of nuclear and plastid DNA sequences support dysploid and polyploid chromosome number changes and reticulate evolution in the diversification of *Melampodium* (*Millerieae*, *Asteraceae*). *Mol. Phyl. Evol.* 53 220–233. 10.1016/j.ympev.2009.02.021 19272456 PMC4268500

[B7] Bloomberg S. P. Garland J. T. Ives A. R. Crespi B. (2003). Testing for phylogenetic signal in comparative data: behavioral traits are more labile. *Evolution* 57 717–745. 10.1111/j.0014-3820.2003.tb00285.x 12778543

[B8] Bouckaert R. Heled J. Kühnert D. Vaughan T. Wu C.-H. Xie D. (2014). BEAST 2: a software platform for Bayesian evolutionary analysis. *PLoS Comput. Biol.* 10:e1003537. 10.1371/journal.pcbi.1003537 24722319 PMC3985171

[B9] Cusimano N. Renner S. S. (2014). Ultrametric trees or phylograms for ancestral state reconstruction: does it matter? *Taxon* 63 721–726. 10.12705/634.14

[B10] Dodsworth S. Chase M. W. Kelly L. J. Leitch I. J. Macas J. Novak P. (2015). Genomic repeat abundances contain phylogenetic signal. *Syst. Biol.* 64 112–126. 10.1093/sysbio/syu080 25261464 PMC4265144

[B11] Dodsworth S. Jang T.-S. Struebig M. Chase M. W. Weiss-Schneeweiss H. Leitch A. R. (2016). Genome-wide repeat dynamics reflect phylogenetic distance in closely related allotetraploid *Nicotiana* (Solanaceae). *Plant Syst. Evol.* 303 1013–1020. 10.1007/s00606-016-1356-9 32009724 PMC6961477

[B12] Edgar R. C. (2004). MUSCLE: multiple sequence alignment with high accuracy and high throughput. *Nucleic Acids Res.* 32 1792–1797. 10.1093/nar/gkh340 15034147 PMC390337

[B13] Felsenstein J. (1985). Phylogenies and the comparative method. *Am. Nat.* 125 1–15. 10.1086/286013

[B14] Felsenstein J. (1993). *PHYLIP: Phylogenetic Inference Package, Version 3.5.* Available online at: http://evolution.genetics.washington.edu/phylip.html (accessed July 14, 2015).

[B15] Fleischmann A. Michael T. P. Rivadavia F. Sousa A. Wang W. Temsch E. M. (2014). Evolution of genome size and chromosome number in the carnivorous plant genus *Genlisea* (Lentibulariaceae), with a new estimate of the minimum genome size in angiosperms. *Ann. Bot.* 114 1651–1663. 10.1093/aob/mcu189 25274549 PMC4649684

[B16] Garrido-Ramos M. A. (2015). Satellite DNA in plants: more than just rubbish. *Cytogenet. Genome Res.* 146 153–170. 10.1159/000437008 26202574

[B17] Harmon L. J. Losos J. B. Davies J. T. Gillespie R. G. Gittleman J. L. Bryan Jennings W. (2010). Early bursts of body size and shape evolution are rare in comparative data. *Evolution* 64 2385–2396. 10.1111/j.1558-5646.2010.01025.x 20455932

[B18] Hawkins J. S. Proulx S. R. Rapp R. A. Wendel J. F. (2009). Rapid DNA loss as a counterbalance to genome expansion through retrotransposon proliferation in plants. *Proc. Natl. Acad. Sci. U.S.A.* 106 17811–17816. 10.1073/pnas.0904339106 19815511 PMC2764891

[B19] Heslop-Harrison J. S. Murata M. Ogura Y. Schwarzacher T. Motoyoshi F. (1999). Polymorphisms and genomic organization of repetitive DNA from centromeric regions of *Arabidopsis* chromosomes. *Plant Cell* 11 31–42. 10.1105/tpc.11.1.31 9878630 PMC144094

[B20] Höhna S. Landis M. J. Heath T. A. Boussau B. Lartillot N. Moore B. R. (2016). RevBayes: bayesian phylogenetic inference using graphical models and an interactive model-specification language. *Syst. Biol.* 65 726–736. 10.1093/sysbio/syw021 27235697 PMC4911942

[B21] Horvilleur B. Lartillot N. (2014). Monte Carlo algorithms for Brownian phylogenetic models. *Bioinform.* 30 3020–3028. 10.1093/bioinformatics/btu485 25053744 PMC4609009

[B22] Kearse M. Moir R. Wilson A. Stones-Havas S. Cheung M. Sturrock S. (2012). Geneious Basic: an integrated and extendable desktop software platform for the organization and analysis of sequence data. *Bioinform.* 28 1647–1649. 10.1093/bioinformatics/bts199 22543367 PMC3371832

[B23] Kelly L. J. Renny-Byfield S. Pellicer J. Macas J. Novák P. Neumann P. (2015). Analysis of the giant genomes of *Fritillaria* (Liliaceae) indicates that a lack of DNA removal characterizes extreme expansions in genome size. *New Phytol.* 208 596–607. 10.1111/nph.13471 26061193 PMC4744688

[B24] Kovařík A. Dadejova M. Lim Y. K. Chase M. W. Clarkson J. J. Knapp S. (2008). Evolution of rDNA in *Nicotiana* allopolyploids: a potential link between rDNA homogenization and epigenetics. *Ann. Bot.* 101 815–823. 10.1093/aob/mcn019 18310159 PMC2710217

[B25] Lysak M. A. Koch M. A. Beaulieu J. M. Meister A. Leitch I. J. (2009). The dynamic ups and downs of genome size evolution in *Brassicaceae*. *Mol. Biol. Evol.* 26 85–98. 10.1093/molbev/msn223 18842687

[B26] Macas J. Meszaros T. Nouzova M. (2002). PlantSat: a specialized database for plant satellite repeats. *Bioinform* 18 28–35. 10.1093/bioinformatics/18.1.28 11836208

[B27] Macas J. Neumann P. Novák P. Jiang J. (2010). Global sequence characterization of rice centromeric satellite based on oligomer frequency analysis in large-scale sequencing data. *Bioinform.* 26 2101–2108. 10.1093/bioinformatics/btq343 20616383

[B28] Macas J. Novák P. Pellicer J. Čížková J. Koblížková A. Neumann P. (2015). In depth characterization of repetitive DNA in 23 plant genomes reveals sources of genome size variation in the legume tribe *Fabeae*. *PLoS One* 10:e0143424. 10.1371/journal.pone.0143424 26606051 PMC4659654

[B29] McCann J. Jang T.-S. Macas J. Schneeweiss G. M. Matzke N. J. Novák P. (2018). Dating the species network: allopolyploidy and repetitive DNA evolution in American daisies (*Melampodium* sect. *Melampodium*, *Asteraceae*). *Syst. Biol.* 67 1010–1024. 10.1093/sysbio/syy024 29562303 PMC6193527

[B30] McCann J. Schneeweiss G. M. Stuessy T. F. Villaseñor J. L. Weiss-Schneeweiss H. (2016). The impact of reconstruction methods, phylogenetic uncertainty and branch lengths on inference of chromosome number evolution in American daisies (*Melampodium*. *Asteraceae*). *PLoS One* 11:e0162299. 10.1371/journal.pone.0162299 27611687 PMC5017664

[B31] Melters D. P. Bradnam K. R. Young H. A. Telis N. May M. R. Ruby J. G. (2013). Comparative analysis of tandem repeats from hundreds of species reveals unique insights into centromere evolution. *Genome Biol.* 14:R10. 10.1186/gb-2013-14-1-r10 23363705 PMC4053949

[B32] Natali L. Cossu R. M. Barghini E. Giordani T. Buti M. Mascagni F. (2013). The repetitive component of the sunflower genome as shown by different procedures for assembling next generation sequencing reads. *BMC Genom.* 14:686. 10.1186/1471-2164-14-686 24093210 PMC3852528

[B33] Novák P. Hřibová E. Neumann P. Koblížková A. Doležel J. Macas J. (2014). Genome-wide analysis of repeat diversity across the family Musaceae. *PLoS One* 9:e98918. 10.1371/journal.pone.0098918 24932725 PMC4059648

[B34] Novák P. Neumann P. Macas J. (2010). Graph-based clustering and characterization of repetitive sequences in next-generation sequencing data. *BMC Bioinform* 11:378. 10.1186/1471-2105-11-378 20633259 PMC2912890

[B35] Novák P. Neumann P. Pech J. Steinhaisl J. Macas J. (2013). RepeatExplorer: a Galaxy-based web server for genome-wide characterization of eukaryotic repetitive elements from next-generation sequence reads. *Bioinform* 29 792–793. 10.1093/bioinformatics/btt054 23376349

[B36] Ogilvie H. A. Drummond A. J. (2016). StarBEAST2 brings faster species tree inference and accurate estimates of substitution rates. *BioRxiv* [preprint]. 10.1093/molbev/msx126 28431121 PMC5850801

[B37] Pagel M. Meade A. (2004). *BayesTraits Manual.* Available online at: www.evolution.rdg.ac.uk/BayesTraits.html (accessed December 12, 2015).

[B38] Pellicer J. Fay M. F. Leitch I. J. (2010). The largest eukaryotic genome of them all? *Bot. J. Linn. Soc.* 164 10–15. 10.1111/j.1095-8339.2010.01072.x

[B39] Plummer M. Best N. Cowles K. Vines K. (2006). CODA: convergence diagnosis and output analysis for MCMC. *R News* 6 7–11.

[B40] Rambaut A. (2007). *Trace**r Version 1.6.* Available online at: http://beast.bio.ed.ac.uk/Tracer (accessed February 22, 2016).

[B41] Rebernig C. A. Weiss-Schneeweiss H. Blöch C. Turner B. Stuessy T. F. Obermayer R. (2012). The evolutionary history of the white-rayed species of *Melampodium* (*Asteraceae*) involved multiple cycles of hybridization and polyploidization. *Am. J. Bot.* 99 1043–1057. 10.3732/ajb.1100539 22645096 PMC4268502

[B42] Renny-Byfield S. Chester M. Nichols R. A. Macas J. (2012). Independent, rapid and targeted loss of highly repetitive DNA in natural and synthetic allopolyploids of *Nicotiana tabacum*. *PLoS One* 7:e36963. 10.1371/journal.pone.0036963 22606317 PMC3351487

[B43] Renny-Byfield S. Kovařík A. Kelly L. J. Macas J. Novak P. Chase M. W. (2013). Diploidization and genome size change in allopolyploids is associated with differential dynamics of low-and high-copy sequences. *Plant J.* 74 829–839. 10.1111/tpj.12168 23517128

[B44] Rudd M. K. Wray G. A. Willard H. F. (2006). The evolutionary dynamics of α-satellite. *Genome Res.* 16 88–96. 10.1101/gr.3810906 16344556 PMC1356132

[B45] Sonnhammer E. L. L. Durbin R. (1995). A dot-matrix program with dynamic threshold control suited for genomic DNA and protein sequence analysis. *Gene* 167 1–10. 10.1016/0378-1119(95)00714-8 8566757

[B46] Stuessy T. F. Blöch C. Villaseñor J. L. Rebernig C. A. Weiss-Schneeweiss H. (2011). Phylogenetic analyses of DNA sequences with chromosomal and morphological data confirm and refine sectional and series classification within *Melampodi*um (*Asteraceae*. *Millerieae*). *Taxon* 60 436–449. 10.1002/tax.602013

[B47] Tek A. L. Song J. Macas J. Jiang J. (2005). *Sobo*, a recently amplified satellite repeat of potato, and its implications for the origin of tandemly repeated sequences. *Genetics* 170 1231–1238. 10.1534/genetics.105.041087 15911575 PMC1451160

[B48] Ugarković Ð (2008). Satellite DNA libraries and centromere evolution. *Open Evol. J.* 2 1–6. 10.2174/1874404400802010001

[B49] Ugarković Ð Plohl M. (2002). Variation in satellite DNA profiles—causes and effects. *EMBO J.* 21 5955–5959. 10.1093/emboj/cdf612 12426367 PMC137204

[B50] Vallès J Canela M. Á Garcia S. Hidalgo O. Pellicer J. Sánchez-Jiménez I. (2013). Genome size variation and evolution in the family *Asteraceae*. *Caryologia* 66 221–235. 10.1080/00087114.2013.829690

[B51] Vu G. T. H. Schmutzer T. Bull F. Cao H. X. Fuchs J. Tran T. D. (2015). Comparative genome analysis reveals divergent genome size evolution in a carnivorous plant genus. *Plant Genome* 8 1–14. 10.3835/plantgenome2015.04.0021 33228273

[B52] Weiss-Schneeweiss H. Blöch C. Turner B. Villaseñor J. L. Stuessy T. F. Schneeweiss G. M. (2012). The promiscuous and the chaste: frequent allopolyploid speciation and its genomic consequences in American daisies (*Melampodium* sect. *Melampodium*; *Asteraceae*). *Evolution* 66 211–228. 10.1111/j.1558-5646.2011.01424.x 22220876

[B53] Weiss-Schneeweiss H. Leitch A. R. McCann J. Jang T.-S. Macas J. (2015). “Employing next generation sequencing to explore the repeat landscape of the plant genome,” in *Next Generation Sequencing in Plant Systematics, Regnum Vegetabile 157*, eds Hörandl E. Appelhans M. (Königstein: Koeltz Scientific Books), 155–180.

[B54] Weiss-Schneeweiss H. Stuessy T. F. Villaseñor J. L. (2009). Chromosome numbers, karyotypes, and evolution in *Melampodium* (*Asteraceae*). *Int. J. Plant Sci.* 170 1168–1182. 10.1086/605876

[B55] Wicker T. Sabot F. Hua-Van A. Bennetzen J. L. Capy P. Chalhoub B. (2007). A unified classification system for eukaryotic transposable elements. *Nature Rev. Genet.* 8 973–982. 10.1038/nrg2165 17984973

[B56] Zozomová-Lihová J. Mandáková T. Kovaříková A. Mühlhausen A. Mummenhoff K. Lysak M. A. (2014). When fathers are instant losers: homogenization of rDNA loci in recently formed *Cardamine schulzii* trigenomic allopolyploid. *New Phytol.* 203 1096–1108. 10.1111/nph.12873 24916080

