# Supplementary material for: Differential Genome Size and Repetitive DNA Evolution in Diploid Species of Melampodium sect. Melampodium (Asteraceae)
Source: Front Plant Sci. 2020 Mar 31;11:362. doi: 10.3389/fpls.2020.00362 (PMC7136903; doi:10.3389/fpls.2020.00362)
Supplement: Supplementary file 1 [file Data_Sheet_1.PDF]

Supplementary Table 1. GenBank accession numbers of sequence data used for phylogenetic analyses (from Blöch et al., 2009, Stuessy et al., 2011 and Weiss-Schneeweiss et al., 2012)

**Species (chromosome base number & ploidy level)**	**GenBank accession numbers**				
	**5S rDNA**	***Pgi*CI**	**PgiCII**	**ITS1-5.8S-ITS2**	***mat*K**
Sect. *Melampodium* ($x = 10$)					
**Ser. *Melampodium***					
M. americanum L. (2*x*)	GU216359-GU216369	GU216452, GU216453	GU216547, GU216551, GU216552	FJ696977-FJ696979	FJ697080, FJ697081
M. diffusum Cass. (2*x*)	GU216402-GU216405	GU216450, GU216451	GU216545, GU216546	FJ696975-FJ696976	FJ697082, FJ697083
M. linearilobum DC. (2*x*)	GU216428-GU216433	GU216442, GU216443, GU216469-GU216471	GU216553, GU216531	FJ696982, FJ696983	FJ697088, FJ697089
M. longipes (A.Gray) B.L.Rob. (2*x*)	GU216406-GU216410	GU216444-GU216446	GU216543, GU216544	FJ696984-FJ696985	FJ697087, FJ697086
M. pilosum Stuessy (2*x*)	GU216344, GU216345	GU216447-GU216449	GU216550	FJ696980-FJ696981	FJ697084, FJ697085
**Ser. *Leucantha***					
M. cinereum DC. (2*x*)	JF277397-JF277404, JF277413-JF277418	JF277503-JF277512	JF277513, JF277533-JF277543, JF277550-JF277564	FJ697006-FJ697008, FJ697014-FJ697017	FJ697101-FJ697107
M. leucanthum Torr. & A.Gray (2*x*)	JF277405-JF277412	JF277483-JF277483	JF277515-JF277532	FJ697003-FJ697005	FJ697108, FJ697109
**Ser. *Glabribracteata***					
M. glabribracteatum Stuessy (2*x*)	GU216370-GU216380	GU216483	GU216514, GU216515	FJ696989	FJ697100
**Ser. *Cupulata***					

M. appendiculatum B.L.Rob. (2*x*)	GU216355-GU216358	GU216500, JF277481	GU216504, GU216509	FJ697030	FJ697116
M. cupulatum A.Gray (2*x*)	GU216319-GU216324	GU216501, JF277482	GU216502, GU216503, GU216507, GU216508	FJ697031, FJ697032	FJ697114, FJ697115
M. rosei B.L.Rob. (2*x*)	GU216340-GU216343	GU216488-GU216491	GU216510	FJ697022-FJ697026	FJ697121, FJ697122
M. tenellum Hook.f. & Arn. (2*x*)	GU216331-GU216339	GU216484-GU216487	GU216506	FJ697027, FJ697028	FJ697117, FJ697118
